# “Worse but Ours,” or “Better but Theirs?” – The Role of Implicit Consumer Ethnocentrism (ICE) in Product Preference

**DOI:** 10.3389/fpsyg.2016.01830

**Published:** 2016-11-22

**Authors:** Dominika Maison, Norbert Maliszewski

**Affiliations:** ^1^Faculty of Psychology, University of WarsawWarsaw, Poland; ^2^Robert B. Zajonc Institute for Social Studies, University of WarsawWarsaw, Poland; ^3^Cardinal Stefan Wyszyñski UniversityWarsaw, Poland

**Keywords:** consumer ethnocentrism, implicit attitudes, IAT, country of origin effect

## Abstract

The goal of this project was to investigate whether consumer ethnocentrism is purely conscious mechanism based on ideology, as suggested by Shimp and Sharma (1987), or rather is an automatic, unconscious process. The aim of the project was an introduction of the Implicit Consumer Ethnocentrism (ICE) concept, measured by the Implicit Association Test (IAT). The goal of the four studies conducted was to investigate the following issues: (a) whether ICE – an automatic mechanism underlying the preference for local products over foreign – this could be observed next to the more ideologically based classic consumer ethnocentrism; (b) what happens when the consumer’s automatic preference for local products (ICE) is confronted by objective evidence of the superiority of foreign products or by the inferiority of local products. It was assumed that ICE could be reduced when foreign products were associated with a higher level of competence than local products, and this could explain the preference for foreign products over local often observed in less developed countries. In study 1 the ICE for different product categories of existing brands was tested, and in study 2 the ICE was measured in the context of non-existent brands. Both studies showed a strong in-group brand preference and confirmed the existence of new phenomena – ICE. The results of studies 3 and 4 again indicated a strong, automatic in-group brand favoritism effect as measured by IAT – participants preferred local brands over foreign. However, the inclusion of well-known foreign brands associated with high competence reduced the IAT effect (in-group preference).

## Introduction

### What is Consumer Ethnocentrism?

The phenomena of preference for local products over foreign is called consumer ethnocentrism and is defined as the consequent and conscious preference for local products over foreign (Shimp and Sharma, 1987; Sharma et al., 1995; Watson and Wright, 2000). Consumer ethnocentrism is usually described as being analogous to an attitude concept; it is an attitude toward local products which contains cognitive, affective and behavioral components. People may believe locally made products are truly better (“Our products have better quality”) (cognitive component), they may have positive feelings for local products (“I love our products”) (affective), and they can behave in an ethnocentric way by purchasing local products (behavioral).

The first research on consumer ethnocentrism was conducted in the United States by Shimp and Sharma (1987). They were focused on understanding this phenomenon and creating a scale to measure the construct called CETSCALE (Consumer Ethnocentric Tendencies Scale). The scale is based on a set of conscious consumer beliefs that buying foreign (in this case not American) products is bad for local industry (i.e., American industry), it has the effect of reducing employment, and therefore is not patriotic behavior. Following these assumptions the majority of items in CETSCALE refer to ideological aspects that it is more appropriate or patriotic to purchase their own country’s products, e.g., “A real American should always buy American-made products,” “Buy American-made products. Keep America working.” The scale is based on 17-items with a seven-point Lickert scale. The research using the CETSCALE showed that this tool could be also used outside North America. Netemeyer et al. (1991) used samples from the United States, France, Japan, and Germany to establish that the CETSCALE was a reliable tool for use in different cultures. The work of Durvasula et al. (1997), using samples from the United States and Russia, both of which are culturally diverse, supported this.

From the research conducted for almost 30 years in many countries we know that consumer ethnocentrism is not only present in American society (Hamelin et al., 2011; Parker et al., 2011; Fakharmanesh and Miyandehi, 2013). However, it does not have the same strength in all countries. Clear preference for domestic products and brands over foreign is observed mostly in well-developed countries (Shimp and Sharma, 1987; Watson and Wright, 2000). A more complicated situation is observed in less developed countries, where many products are not manufactured locally, and those that are often of objectively inferior quality. In those countries consumers usually have a weaker preference for local products and a stronger preference for foreign (Bilkey and Nes, 1982; Batra et al., 2000; Watson and Wright, 2000; Tsai et al., 2013). This does not mean that consumer ethnocentrism is limited to Western countries, but its intensity depends on the level of economic development of the country.

Beyond economic factors (levels of country development), consumer ethnocentrism depends also on demographic, psychological and cultural variables. The majority of research investigating the role of demography in consumer ethnocentrism shows a positive correlation with age (older people are more ethnocentric) and a negative correlation with education and wealth (more highly educated people and those in a better financial situation are less ethnocentric – Bawa, 2004; Nadiri and Tümer, 2010). Yoo and Donthu (2005) investigated the relationship between consumer ethnocentrism and individualism vs. collectivism (Hofstede, 1984). In the study conducted in the United States consumer ethnocentrism correlated with collectivism and individualistic people were more open to foreign products (Yoo and Donthu, 2005). Other psychological variables that were found to correlate with consumer ethnocentrism are patriotism, conservatism (Anderson and Cunningham, 1972; Han, 1988) and nationalism (Tsai et al., 2013). Research done in Turkey showed that devoted Muslims, having a stronger social identity and feeling a strong sense of national belonging, have higher levels of consumer ethnocentrism than non-Muslims (Kaynak and Kara, 2002). In a study conducted in Poland consumer ethnocentrism was found to be correlated with Catholicism, traditionalism and patriotism (Adamczyk et al., 2015) and all dimensions of social identity measured with the Cameron scale (Cameron, 2004) and with the self-perception of respondents being patriotic (Maison and Baran, 2014).

Preference for local vs. foreign products is a complicated and multidimensional phenomenon and the results depend on the way of measuring it. A survey conducted in Poland in 2000 (representative national wide sample *N* = 1005) showed that the majority of Poles (65%) declare that while shopping they prefer and choose Polish products over foreign products; only 15% declared a preference for foreign products over Polish (20% declared they had no preference) (Maison, 2004). However, the situation was more complicated when the survey questions became less general and asked about preferences for local or foreign in different product categories. In the case of food and drinks consumers declared a preference for Polish products, but in the case of durables (e.g., cars, electronics, clothes) they declared a preference for foreign products. These results might be a consequence of current (or past, in the case of Poland) bad experience with the quality of local products, especially in the case of products where production requires a high level of technological development (Batra et al., 2000; Watson and Wright, 2000). The exceptions are food products with local produce being perceived as more natural and containing fewer preservatives.

We get an even more complicated picture when we observe market shares of products with different countries of origin. In the case of Poland, an analysis of the market share of different brands of cosmetics conducted at the same time as the above mentioned survey showed sales of foreign brands were higher than local brands. In 2000 74% of sodas sold were foreign brands, and only 26% were Polish (based on the value of sales). A similar proportion was observed in beauty products: foreign brands of shampoo had a 77% market share vs. 23% for Polish brands; foreign hair care products had an 83% market share vs. 17% for Polish products (source MEMRB – in-store panel provider). When analyzing data from market share analyses and survey results based on different questions we can observe a very complex and ambivalent picture of consumer ethnocentrism and a dissociation between declared preferences and the actual purchase. However, this depends on the product category and on how detailed a question is asked (on a category or brand level). In the case of food products people buy more local products but in the case of durables, electronics – more foreign. Moreover, we have to remember that consumer ethnocentrism is just one of the factors which influence a purchase decision, next to price, quality, availability in the shop and product features.

### Consumer Ethnocentrism – A Reflective or Automatic Phenomenon

Shimp and Sharma (1987) assumed that consumer ethnocentrism has a strong moral and ideological background and therefore CETSCALE was constructed based on a set of conscious consumer beliefs. The main goal of our research project was to look at the phenomenon of consumer ethnocentrism from a different, psychological perspective, and investigate whether it is, as Shimp and Sharma (1987) suggested, only an ideology based conscious mechanism, or also has another side – based on automatic and unconscious beliefs. We suggest that the source of this phenomenon is both moral/ideological and psychological, and next to the deliberated mechanism, can also be derived from automatic and unconscious mechanisms, such as in-group favoritism bias (Tajfel, 1978), which is manifested as an automatic preference for “our” group. Based on this basic psychological mechanism, products manufactured in one’s own country are automatically perceived favorably because they are “ours” and not “theirs.” Therefore we assume that consumer ethnocentrism, next to conscious, deliberative opinions, can be also based on automatic “in-group brands favoritism” and, independent of conscious evaluation, local products can be treated in a more favorable way than foreign on an automatic level. And, as a consequence, in some situations a consumer will choose foreign and in others local products, regardless of his deliberated preference.

In our studies we introduced the concept of Implicit Consumer Ethnocentrism (ICE), defined as a strong and *automatic preference* for local products over foreign. This phenomenon has the character of a primary mechanism, which, depending on the consumer experience, can be supported by market reality (as in highly developed countries) or can be suppressed by the market reality (as in less developed countries). To measure ICE we adapted the *Implicit Association Test* (IAT) – a reaction time (RT)-based method to study automatic implicit attitudes (Greenwald and Banaji, 1995).

### A New Approach to Attitudes – Automatic and Beyond Conscious Control

Over the last 30 years there can be observed a marked evolution in understanding an attitude concept in psychology. The predominant way of understanding it was as being comprised of cognitive, emotional and behavioral factors. These factors refer to thoughts, feelings, and behavior toward an attitude object (Allport, 1935). This approach assumed that a person was consciously aware of his or her attitude and could put it into words and express it in a questionnaire. Moreover, it also assumed that the three mentioned components (cognitive, affective, and behavioral) are correlated and can predict behavior. However, many psychological studies (including consumer psychology) show a weak correlation between these three attitude components and a weak prediction of behavior (Fazio and Zanna, 1981; Kraus, 1995).

In response to problems with this traditional attitude concept, and the difficulties inherent in measuring it based on introspection and declaration, psychologists began to look for new attitude research methods. In the 90s two new concepts of attitudes were introduced in psychology: “dual attitudes” (Chaiken and Trope, 1999) and “implicit attitudes” (Greenwald and Banaji, 1995). The basis of the dualism of attitude concept is the assumption that a person can at the same time hold two different attitudes toward an object, a conscious attitude and another attitude of which the person is not consciously aware (Chaiken and Trope, 1999). In turn implicit attitude is defined as the traces of past experiences, even ones no longer remembered, and cannot be accurately introspectively identified, it may also influence reactions (Greenwald and Banaji, 1995).

A consequence of understanding attitudes in this new way, as an implicit and automatic processes, was that psychologists needed to find new methods to study them because tools based on declaration and introspection were not particularly useful when looking at processes which are uncontrolled and inaccessible to consciousness. The first change was a move away from the self-descriptive questionnaire and respondent declarations, and more indirect measures were employed. With indirect measures the outcomes are independent of participants’ conscious control as they are unaware of what is being measured. The second change was the introduction of RT-based methods, in which the time taken to respond is an indicator of attitude (Gaertner and McLaughlin, 1983; Fazio et al., 1986; Lalonde and Gardner, 1989; Fazio, 1990; Zárate and Smith, 1990; Bargh, 1999). Most of the methods using RT rely on the assumption that faster RTs indicate the target object (object of attitudes) and relevant attributes (describing this object) are more strongly associated. Shorter RTs imply a stronger implicit attitude toward the object (Fazio, 2001). Presently, there are available several methods using RT to study implicit processes, for example, *Lexical Decision Task* (Wittenbrink et al., 1997), *Adjective Evaluation Task* (Fazio et al., 1986), *Category Inclusion Task* (Dovidio et al., 1986), *Extrinsic Affective Simon Task* (EAST; De Houwer, 2003), the *Implicit Relational Assessment Procedure* (*IRAP*; Barnes-Holmes et al., 2010), and IAT (Greenwald et al., 1998). The latter is arguably the most well-known and widely used method.

### Implicit Association Test – Background for Implicit Consumer Ethnocentrism (ICE)

The IAT is a method of studying implicit attitudes introduced by Greenwald and Banaji (1995). It is a computer-based categorization task designed to measure relative strengths of associations between concepts in memory. Initially IAT research focused on measuring a racial implicit attitude (Greenwald et al., 1998; Dasgupta and Greenwald, 2001; Greenwald and Nosek, 2001), self-concept (Farnham et al., 1999; Greenwald and Farnham, 2000; Greenwald et al., 2002), self-esteem (Farnham et al., 1999; Greenwald et al., 2002), gender stereotypes (e.g., Rudman et al., 2001), and stigmatized behavior such as smoking (e.g., Swanson et al., 2001). The IAT is now also used as a tool to research consumer attitudes (Maison et al., 2001, 2004; Perkins et al., 2008; Maison and Gregg, 2016). IAT interpretation is based on the assumption that it is easier to give the same response to items belonging to two categories when those categories have congruent evaluation (e.g., items perceived as pleasant are paired with pleasant words) than when they are not congruent (e.g., items the participant perceives as pleasant are paired with unpleasant words). In the IAT task participants are unaware of the associations being assessed, or their strength, and it is therefore described as an implicit measure. Furthermore, the IAT is carried out under time pressure and is not easily faked, so it is assumed to capture automatic reactions rather than consciously considered reactions (Röhner et al., 2011).

**FIGURE 1:**
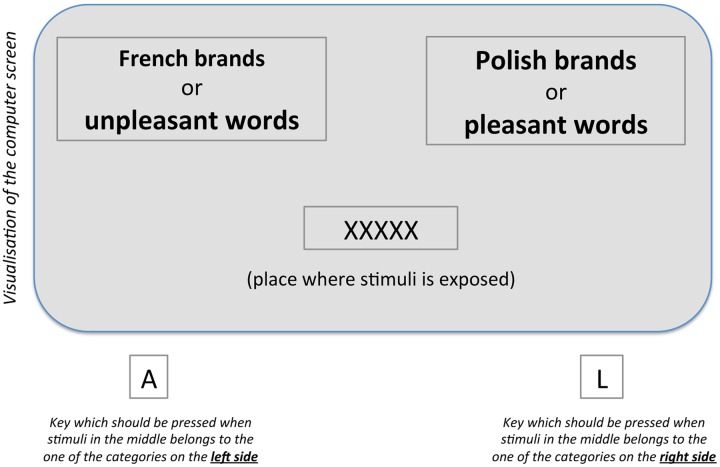
**Example of the Implicit Association Test (IAT) to measure implicit attitudes toward Polish and French brands**.

The IAT method involves measuring the time a participant takes to complete computerized categorization tasks. The participant is presented with a stimulus, which he or she is required to place into one of two groups (for example in study 3: Polish vs. French brands). Pleasant and unpleasant words are the standard attribute categories. Three of the seven blocks in the IAT task are *single categorization* tasks, which are practice tasks where the participants are exposed to stimuli and learn to respond appropriately. The other four blocks are *combined tasks*, where four categories are categorized simultaneously (e.g., French, “unpleasant” words and Polish, “pleasant”); the time taken to categorize stimuli associated with the attitude object and attributes is measured (two blocks are training blocks and two are crucial tasks for the analysis). As the participant performs the crucial task, the name of one of the target categories and the name of one of the attribute categories are presented (e.g., “French” and “unpleasant” on the left side of the computer screen and “Polish” and “pleasant” on the right – see **Figure 1**). While this is happening, words belonging to each of four categories are shown randomly in the middle of the computer screen, one at a time (e.g., names of Polish brands, names of French brands, positive and negative words). The participants’ task is to press (as quickly as possible) either the left- or right-hand key (often it is the “A” and “L” key), depending on the side of the screen on which the label of target or attribute category is displayed (e.g., “Polish,” “French,” “pleasant,” “unpleasant”) corresponding to the stimuli appearing in the middle. Two crucial *combined tasks* differ on the side of the screen, on which are displayed the names of categories of attitude object (French and Polish). These tasks are known as the *initial combined task* and the *reversed combined task.* In the initial combined task “French” and unpleasant words appeared on the left side of the screen while “Polish” and pleasant words appeared on the right (see **Figure 1**). For the reversed combined task it was the opposite: participants were shown “Polish” and unpleasant words on the left side and “French” and pleasant words on the right side. In the standard IAT procedure the order of the combined tasks are always counterbalanced over participants. It means that one participant starts with one combination (e.g., “French” and “unpleasant words” on one side and “Polish” and “pleasant words” on other side) followed by the reversed task, but the other participant is doing the opposite order, starting with the second combination (e.g., “Polish” and “unpleasant words” on one side and “French” and “pleasant words” on the other side) followed by the reversed task.

Participants press the response key (“A” or “L” in the example above) in order to categorize the stimuli into the appropriate group. The task should be performed as quickly as possible. In interpreting the IAT it is assumed the ease with which a person is able to assign the same response to distinct concepts is related to the strength of the association between those concepts. The underlying assumption is that people are able to give the same response to items in two categories more easily when those categories have the same evaluative value (e.g., both are positive or negative) than when they are not (e.g., one is positive and one is negative). The analysis is based on RTs from combined tasks. The difference in average response time for the initial combined task and the reversed combined task is an indicator of the IAT effect.

Many conducted studies using IAT proved that it is a very useful method to research more complex attitudes, especially when the attitude object is ambivalent, sensitive to impression management or contains a strong automatic component. However, it also proves that the IAT effect might be influenced by other factors than just implicit attitudes, e.g., familiarity effect (Brendl et al., 2001), public attitudes instead of individual (Han et al., 2010), and that sometimes the IAT reflects, not underlying associations, but only superficial overlaps in category salience (Rothermund and Wentura, 2004). Therefore, researchers who have to use this method should interpret the results with caution (Maison and Gregg, 2016).

## Implicit Consumer Ethnocentrism – The Goal of the Research Project

In our studies we applied the IAT to test ICE. We assumed that favoritism of local products could be observed not only based on declaration, but also on the level of automatic implicit preferences, as measured using the IAT. We tested ICE on different product categories of existing brands (study 1) and checked whether this automatic process was also observed for new, non-existent brands (study 2). In the research project we also investigated the extent to which the automatic ICE can be modified with market experience, and when local products are perceived as having inferior or superior quality to foreign products (studies 2–4).

## Implicit Consumer Ethnocentrism (ICE) vs. Explicit Brand Preference for Different Product Categories of Existing Brands (Experiment 1)

The goal of Experiment 1 was to explore the phenomenon of ICE – the automatic preference of local products over foreign. It was expected, that even though in non-western countries consumers on an explicit level prefer foreign products over local and more often choose foreign brands, on the implicit level they might express automatic implicit preferences for local brands, as measured using the IAT. It was expected that the IAT task would be performed faster than the opposite task when Polish brands were categorized on the same side of the computer screen as positive words. This means that the IAT effect would show automatic favoritism for local brands and that a discrepancy between explicit and implicit brand preference depending on their origin would be observed. Even though most people will explicitly prefer foreign brands, implicitly they will have a preference for local brands (as a consequence of in-group preference, which is automatic).

### Method

#### Ethics Statement

We used the standard IAT task procedure (https://implicit.harvard.edu/implicit/takeatest.html), where at the beginning of the computer task participants are asked to participate in the study and are told that they can stop the task at any moment. At the beginning of the study participants were also informed that the study is anonymous and the data will be analyzed at a group level (not individual). Clicking a button indicated consent. This study was carried out in accordance with the recommendations of the Robet B. Zajonc Institute for Social Studies’ ethics committee with informed consent from all subjects. All subjects gave informed consent in accordance with the Declaration of Helsinki. The protocol was approved by the Robet B. Zajonc Institute for Social Studies’ ethics comittee.

#### Participants

Ninety two Polish college students, 47 women and 45 men, aged 18–19 (*M* = 18; *SD* = 1.91) participated in the research.

#### Research Methods and Materials

The study used an IAT task consisting of a standard set of attributes: five positive (joy, smile, fun, vacation, happiness) and five negative (death, disease, murder, vomit, poison). The target stimuli used in the study were names of Polish and foreign brands present at that time on the Polish market. The brands belonged to different product categories, both FMCG and durables. In each product category two brands were chosen, of which one was foreign and one was Polish: (1) cigarettes: Marlboro vs. Sobieskie; (2) vodka: Absolut vs. Żytnia; (3) car: Ford vs. Polonez; (4) sodas: Pepsi vs. Hoop; (5) sports equipment: Reebok vs. Polsport; (6) electronics: Sony vs. Unitra; (7) household equipment: Whirlpool vs. Polar; (8) toothpaste: Signal vs. Colodent. Brands were chosen based on a pilot study where there was controlled brand awareness and perception whether the product was Polish or foreign. For the study we chose only brands for which there was a high awareness and clear perception of origin.

The BPM method (*Brand Preference Measure*, Maison and Baran, 2014) was used as an explicit measure of Polish vs. foreign brand preference. From eight pairs of brands (of which one element of the pair was Polish and the other foreign) belonging to eight different product categories, the respondent had to point out the one he/she is using or prefer. The method was used to determine brands’ closeness to the consumer. The idea behind the method is its simplicity therefore the task of the respondent is a forced choice of one brand from a pair of brands. Pairs of brands in each category are selected based on the pilot study is such a way, that the most important difference between brands in a pair is their origin (other variables such as awareness or availability are controlled and equalized). The results are analyzed on the global level of total preference of brands belonging to one or other category (in this case Polish or foreign) and not at the level of each pair (when in each case other factors might interfere). The indicator of consumer ethnocentrism is a summarized number of Polish brands chosen by each respondent. The BPM result has relative value – it can be compared only within the sample where it was used, because its value depends on the brands selected for particular study. The brands chosen for this task were the same as used in the IAT task.

#### Procedure

As a first task participants filled in a BPM where the preference for different brands was measured. After that the IAT task was conducted, where four categories were used: (a) target categories – the same set of Polish and foreign brands as used in the explicit part (BPM); (b) attribute categories – a set of positive and negative words. The order of IAT and BPM was not counterbalanced over participants. The IAT task was performed second because it is much more difficult to be influenced by other measures than explicit measures (Kim, 2003; Röhner et al., 2011).

### Results

The analysis based on the BPM task revealed a marked explicit preference for foreign brands as opposed to Polish brands. From eight pairs of brands the average number of foreign brands chosen in the sample was 6.95 (Median for foreign brands is 7 and for Polish is 1). Only 9% of respondents chose Polish brands more often than foreign, while 91% more often chose the foreign brands. This shows strong explicit preference for foreign brands over Polish brands.

Comparison of the two IAT tasks (categorization time when Polish brands were at the same side with positive words and foreign brands with negative words, with the opposite task) showed the significant difference, *F*(1,91) = 15.95; *p* < 0.001, η^2^ = 0.15. The IAT task when Polish brands were at the same side with positive words and foreign brands with negative ([-]Foreign/Polish[+] – *M* = 841 ms.; *SD* = 186) was performed faster than the opposite task ([-]Polish/ Foreign[+] – *M* = 924 ms.; *SD* = 229). Contrary to the explicit brand preference the IAT showed Implicit Consumer Ethnocentrism (ICE) – an automatic preference for Polish brands over foreign.

**FIGURE 2:**
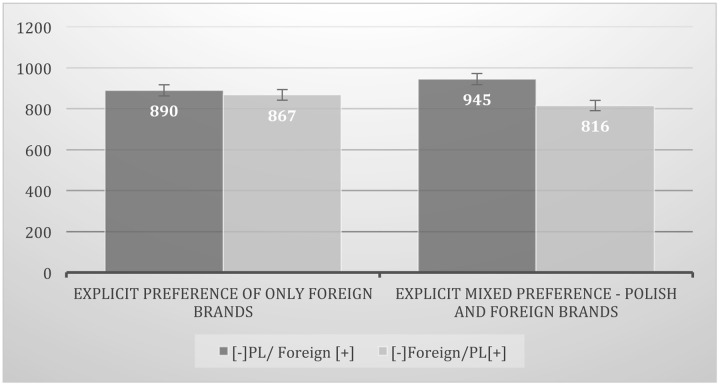
**Comparison of Implicit Consumer Ethnocentrism (ICE) toward Polish vs. foreign brands (IAT effect) among people who prefer on explicit level:** (a) only foreign brands and (b) those with mixed preference for foreign and Polish brands. Categorization time in milliseconds (ms).

At the next step of the analysis, the IAT effect (the time difference between two tasks) was compared for people showing different levels of explicit preference for Polish brands in the BPM task (explicit ethnocentrism). To conduct this analysis participants, based on the data distribution, were split into two subgroups of different levels of brand preference: (a) those preferring only foreign brands in each pair (all eight foreign brands chosen – 42% of the sample) and (b) the rest – those who preferred some Polish and some foreign brands (“mixed preference” – 58% of participants). The analysis of variance (mixed mode with repeated measures) was conducted: 2 (explicit preference: “only foreign brands” vs. “mixed”) × 2 (IAT task [RT]: [-]PL/ Foreign[+] vs. [-]Foreign/ PL[+]). Comparison of the IAT effect between two groups with different levels of explicit ethnocentrism (those consumers who in explicit preference in all pairs preferred foreign brands and those who preferred at least some Polish brands) showed a significant difference between those two groups of respondents in the IAT effect, *F*(1,90) = 6.52; *p* = 0.01, η^2^ = 0.07 (see **Figure 2**). The ICE was observed only among those who had mixed explicit preference (some level of explicit ethnocentrism) [*t*(52) = 4.53; *p* < 0.0001], but not in the group of consumers who on an explicit level preferred only foreign products; *t*(38) = 1.02; *p* = 0.3.

### Discussion

The results of Experiment 1 showed a clear explicit preference for foreign brands. The majority of the respondents, when performing the BPM task, were expressing a preference for global brands and not local (Polish ones). This confirmed predictions and also observations from the previous studies, that Polish consumers explicitly prefer foreign brands over local (Maison and Baran, 2014; Adamczyk et al., 2015).

The comparison of IAT effects showed, on the contrary, a stronger automatic preference for Polish brands (the same brands as used in BPM). Polish brands were much more quickly categorized when they were at the same side of the screen with positive words than when they were at the same side with negative words. This result confirmed the second prediction showing the existence of ICE. The results showed also that ICE was observed only among those who at the explicit level preferred at least some Polish brands (even though the majority of them still explicitly preferred mostly foreign brands). Only among those consumers who explicitly preferred exclusively foreign brands (they didn’t mention the Polish brand in any of the pairs) ICE was not observed (IAT effect was not present).

This study delivers new insights into knowledge about consumer ethnocentrism. The results reveal consumer ethnocentrism to be a complex phenomenon. We observed the discrepancy between explicit (declared) and implicit preference (IAT): respondents declared their preference for foreign brands, but on implicit level Polish brands were favored. The different origins of explicit and implicit attitudes toward brands may explain this. While it is likely explicit attitude is formed based on rational elaboration and experience, implicit attitudes are based on emotional cues, which includes having an automatic positive response to in-group (in this case Polish) brands.

Looking at the results showing so strong a preference for Polish brands over foreign (in contrast to explicit preference) one might ask if the observed IAT effect is not a consequence of factors other than only consumer ethnocentrism, such as language or familiarity. Though, all foreign brands used in the study have high brand awareness and are advertised in Poland. Therefore their visibility is high. Moreover, the spelling of some foreign brands is not following Polish rules (e.g., Hoop, Colodent), therefore also the language issue is probably not a valid explanation of ICE effect.

Another question, which arises in the context of the data presented above, is: to what extent is the observed effect of shortened response time related to brand origin (e.g., connected to brand image)? Is there a more fundamental characteristic – not directly related to the brand origin – connected to automatic in-group favoritism (Tajfel, 1978)? Many studies on the minimal group paradigm showed that a random division of people into two groups, for example possessing a red or green pen was enough to create an in-group favoritism reaction (Tajfel, 1978). Ashburn-Nardo et al. (2001) showed that even a random assignment of respondents to groups labeled as preferring one of two fake painters (Quan or Xanthie) was enough to observe implicit preference for one or other painter in the IAT task. For example, those who were labeled “preferring Quan” also expressed stronger implicit preference for Quan than Xanthie. Because the “preference” label for Quan or Xanthie was assigned randomly, it had nothing to do with actual preference for a particular painter but had a much more basic character. We assume that such automatic implicit preference can also be observed in the context of brands. Local products, independently of experience with brands, can be on an implicit level favored as “ours” (national, local) as opposed to foreign, categorized as “other.”

## Implicit Consumer Ethnocentrism (ICE) vs. Competence of Country of Origin – Research on Non-Existent Brands (Experiment 2)

The main question for the next study was to examine to what extent the effect observed in Experiment 1 was connected to the origin of brands derived from an existing brand image. Did the effect actually have a rather more primary nature, connected to the basic effect of categorization? If this ICE is based on such basic mechanism, it should be possible to create an ICE effect with unknown, faked brands, analogous to the implicit preference seen for non-existent painters (Ashburn-Nardo et al., 2001). Therefore in Experiment 2 ICE for non-existent brands was investigated.

The second question of this study was connected to the country-of-origin effect (COE). The COE effect is defined as the positive or negative influence of the country of origin (country of production) of the product or brand in terms of attitude toward it and probability of buying it (Obermiller and Spangenberg, 1989; Elliott and Cameron, 1994; Verlegh and Steenkamp, 1999). Many researches on the country-of-origin effect have shown that providing consumers with information about the origin of the product or brand influences information processing (Hong and Wyer, 1989; Maheswaran, 1994). When the consumer believes that particular country is a specialist in producing that particular type of merchandise, information that this product comes from this country increases the positive perception of this product. And the opposite is also true – a belief about lack of expertise can create a negative perception of a product manufactured in this country. In a study about the perception of cars based on country-of-origin (Maison, 2009) information about the origin of the same car seen in a photo (French or German-made) influenced the car’s perception consistent with the stereotype of that country: i.e., the car was seen as having better esthetics (French origin) or as being more functional (German origin). In a study by Maheswaran (1994), when respondents were provided with powerful arguments about a product (a PC computer), information about the country of origin (Japan vs. Taiwan) did not affect its perception, but when provided with weak arguments, the country-of-origin did affect how the PC was viewed. The mechanism behind how the country-of-origin information works is similar to how stereotypes work. Following this reasoning, we assumed that when products of Polish origin are contrasted with other brands of foreign origin, however, not described in a general way as “foreign,” but as coming from a particular country (activation of COE), this could have an influence on ICE and modify an automatic tendency for favoring in-group products. Moreover, we assume that contrasting Polish (in-group) brands with brands produced in another country will have different effects depending on the product category – whether this other country is perceived as being a specialist or not in this product category. If the comparison refers to a category in which the out-group is perceived as competent it can decrease ICE. On the other hand, contrasting Polish (in-group) brands with brands belonging to a category in which the out-group is not perceived as competent can strengthen ICE or leave it unchanged. Therefore, the second main goal of our studies was to verify whether ICE could be reduced by the *country-of-origin effect* when foreign products were associated with higher competence than local products.

In this experiment each respondent conducted one from two IAT tasks where (non-existent) Polish origin brands were contrasted with (non-existent) foreign origin brands belonging to two categories of products: (a) product category in which the other country (contrasted in the IAT test) is perceived as being a more competent producer than Poland; (b) product category in which the other country (contrasted in the IAT test) is perceived as a less competent producer than Poland.

### Pilot Studies – Choice of Product Categories (Depending on Competence Level) and Names of Fiction Brands

In order to choose product categories in which the out-group is perceived as less or more competent than Poland, and to choose fake brands belonging to those product categories, we conducted two pilot studies. The goal of the first pilot study was to choose the out-group country and two product categories in which: (a) Poland is perceived as a better (more competent) producer than this country and (b) Poland is perceived as an inferior producer to this country. Participants (*N* = 75 students) evaluated the competence of Polish, French and German producers in four different product categories (cheese, meat, beer, and wine) using a seven-point scale. Statistical analysis (*t*-test for independent sample) showed that the biggest differences in perception of competence were observed in the case of comparisons between Poland and France (comparisons between Poland and Germany gave smaller differences). Therefore France was chosen as a reference country for the main study. The biggest difference in levels of competence between Poland and France were perceived in two categories: (a) wine – where France was perceived as a more competent producer than Poland [France *M* = 6.72; *SD* = 0.51; Poland *M* = 3.64; *SD* = 1.15; *t*(74) = 21.35; *p* < 0.001] and (b) beer – where Poland was perceived as a more competent producer than France [France *M* = 3.55; *SD* = 1.26; Poland *M* = 5.87; *SD* = 0.95; *t*(74) = 12.94; *p* < 0.001]. Based on those results wine (France more competent than Poland) and beer (Poland more competent than France) were chosen as the product categories to be tested in two IAT tasks.

The purpose of the second pilot study was to choose exemplars (fake brand names) for the two IAT tasks (wine and beer). The assumption was that the country of origin (Poland or France) of all fictional brand names should be clear and possible to decode correctly. Moreover, each chosen brand name should be perceived as equally suitable for both categories: a wine and beer brand name. For the pilot study we created 10 non-existent Polish and 10 non-existent French brand names. In the case of each tested brand respondents (*N* = 58 students) had to answer three questions referring to two product categories: (1) is this a good name for a new beer/wine?; (2) what quality of product (wine and beer) did they expect based on the brand name?; (3) to what extent did the brand name fit the product category (wine or beer)? From 20 names tested, we chose four brand names which were definitely recognized as Polish: *Miekińskie*, *Szlacheckie*, *Od Cystersów*, *Od Benedyktynów*, and four brand names recognized as having French origin: *Aquitaine, Nobliau, Monastére*, *Bénédictin*. At the same time those were brand names with a minimum difference in evaluation as being suitable for beers and wines (equally suitable to both categories). Moreover, all chosen brand names evoked comparable product quality expectations (no significant differences between brand names).

### Method

#### Participants

The participants in the experiment were 30 male students from the University of Warsaw, aged 22–32 years (*M* = 24.14; *SD* = 1.91).

#### Research Methods and Materials

The same basic IAT method as described in Experiment 1 was applied to measure ICE: the same number of trials and the same standard set of five positive and five negative stimuli were used. The stimuli in the target categories (wine and beer) were names of four fictional Polish and four fictional French brands for both the wine and beer IAT tasks.

#### Procedure

Each respondent performed the IAT task individually on the computer. Participants were randomly assigned into one of two groups. One group (50%) carried out the IAT task with wine as a target category while the other group (50%) did the task with beer as a target category.

### Results

We expected that in the case of the product category where Poland is perceived as more competent (beer) than the foreign country (France) an ICE effect would be observed. We also expected a reduced ICE effect in the case of the product category where the foreign country was considered to be of higher competence (wine).

**FIGURE 3:**
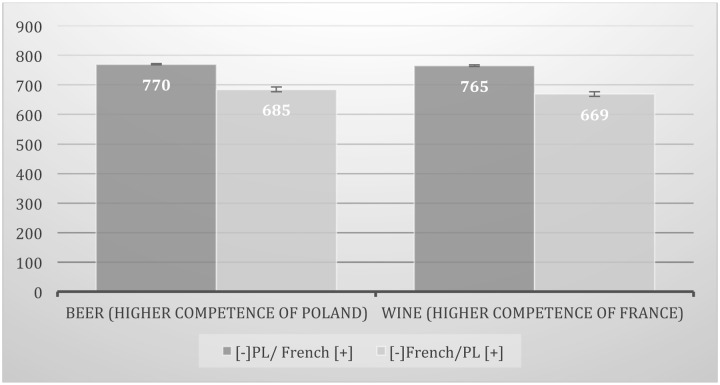
**Comparison of ICE toward Polish (Polish) vs. French (foreign) non-existent brands (IAT effect) in two product categories of different level of competence:** (a) beer – higher competence of Poland and (b) wine – higher competence of France. Categorization time in milliseconds (ms).

To verify these predictions the analysis of variance (mixed mode with repeated measures) was conducted: 2 (IAT task [RT]: [-]PL/FR[+] vs. [-]FR/PL[+]) × 2 (product category: beer and wine IAT). The analysis showed a significant difference in RT between two IAT tasks (significant IAT main effect): RT when Polish brands were categorized at the same side of the computer screen with negative words and French with positive ([-]PL/FR[+]) was significantly longer (*M* = 768; *SD* = 139) than when the opposite task ([-]FR/PL[+]) was performed [*M* = 677; *SD* = 156; *F*(1,28) = 15.34; *p* < 0.001, η^2^ = 0.35]. However, the main effect of product category [wine and beer; *F*(1,28) = 0.04; *p* = 0.835; η^2^ = 0.002] and interaction effects were not significant, *F*(1,28) = 0.04; *p* = 0.835; η^2^ = 0.002 (see **Figure 3**).

### Discussion

The results of Experiment 2 confirmed the existence of ICE – an automatic favoritism of local brands over foreign. Respondents categorized Polish brands faster than French when they were paired with positive words rather than with negative. In this experiment, in contrast to Experiment 1, the effect was observed in the context of non-existent brands, which means that the effect is deep and has an automatic character. It is based more on the basic mechanism of in-group favoritism than on real knowledge about brand image or experience with brands.

The results of the study showed also, that the ICE effect is independent from the out-group’s (in this case France’s) perceived level of competence in particular product categories. In both product categories, when France was perceived as more competent producer than Poland (wine) and when it was perceived as less competent (beer), the ICE effect revealed strong implicit favoritism of Polish brands. In other words, the study didn’t show the expected difference in ICE depending on the competence of the producer. When Polish brands were compared independently with French ones in categories where Poland is perceived as competent (beer), or France is perceived as superior producer (wine), the same ICE effect was obtained. In cases of both product categories RT showed implicit preference for Polish brands, therefore we didn’t observe an expected influence of out-group competence on ICE. However, because Experiment 2 was conducted with non-existent brands, we need to question what in fact caused the ICE effect. Was it a preference of Polish brands or just the familiarity of the brand’s names (effect caused by linguistic familiarity of the stimuli, not consumer effect)? Brendl et al. (2001) showed that fictional words (unknown to anybody) were categorized more quickly and easily when they were teamed with negative words rather than with positive. It is interesting that this effect was sustained even when the contrasted category was insects (category with strong negative evaluation).

## Implicit Consumer Ethnocentrism (ICE) vs. Competence of Country of Origin – Research on Existing Brands (Experiment 3)

The goal of Experiment 3 was to replicate Experiment 2, but in this case with existing brands. We suspected that lack of significant effect of country competence in Experiment 2 was a consequence of using faked brands as stimuli. We wanted to check whether a difference in ICE depending on the competence of the country of origin (in this case France) would be observed also when we use existing brands. We expected, similarly to the Experiment 2, that when French brands were contrasted with Polish in the category where Poland has higher competence (beer) we would observe typical ICE. However in the case of categories where France is perceived as having higher competence (wine), we expected the ICE effect would be minimized or even reversed.

### Method

#### Participants

The participants in this experiment were 30 male students from the University of Warsaw, aged 19–25 years (*M* = 21,4; *SD* = 1,47). The study was conducted only with male participants because men in Poland are drinking alcohol (and especially beer) more frequently than women. Therefore we assumed they are more familiar with alcohol brand names.

#### Research Methods and Materials

Implicit Consumer Ethnocentrism was measured by the IAT method, as described in previous experiments. The target stimuli used in the study were names of existing Polish or French brands of beer and wine. As exemplars of beer were chosen the following brands: (a) Polish: Tyskie, Żywiec, Lech, Żubr; (b) French: Jenlain, La Reserve, Fischer, Desperados. As exemplars of wine were chosen the following brands: (a) Polish: Herbowe, Ciemny Burgund, Dzban leśny, Staropolskie; (b) French: Bongeronde, Bordeaux A.O.C, Meursault, Château Saint Cosme.

#### Procedure

Each respondent performed one of two IAT tasks (wine or beer) individually on the computer. Similarly to previous studies participants were randomly assigned into groups using one of the experimental conditions: (a) with wine as a target category or (b) with beer as a target category. Half of them accomplished the IAT version with wine as a product category and the other half with beer.

### Results

To verify the predictions the analysis of variance (mixed mode with repeated measures) was conducted: 2 (IAT task [RT]: [-]PL/FR[+] vs. [-]FR/PL[+] vs. × 2 (product category: beer vs. wine). The analysis showed a significant difference in RT between two IAT tasks (IAT main effect): RT when Polish brands were categorized at the same side of the computer screen with negative words and French with positive ([-]PL/FR[+]) was significantly longer (*M* = 700; *SD* = 85) than when opposite task ([-]FR/PL[-]) was performed [*M* = 652; *SD* = 64; *F*(1,28) = 6.56; *p* < 0.05; η^2^ = 0.19]. This results shows that respondents expressed ICE in favor of Polish brands. The main effect of product category (beer, *M* = 652; *SD* = 58; wine, *M* = 699; *SD* = 72) was also significant *F*(1,28) = 6.29; *p* < 0.05; η^2^ = 0.18.

**FIGURE 4:**
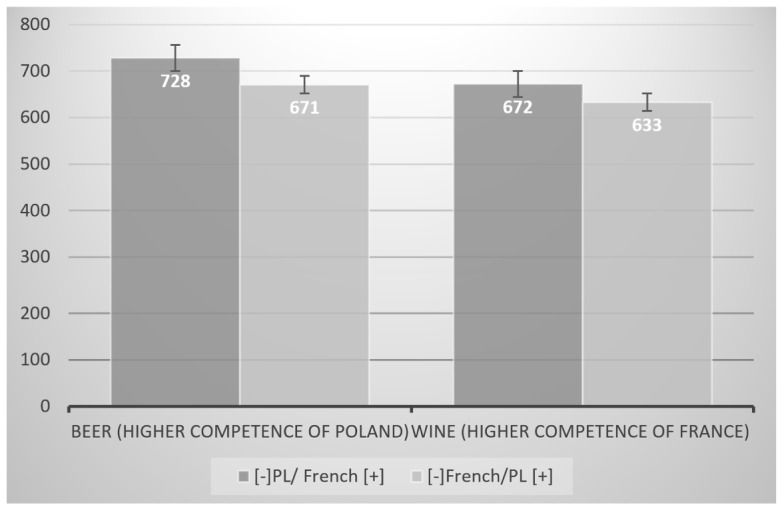
**Comparison of ICE toward Polish (local) vs. French (foreign) existing brands (IAT effect) in two product categories of different levels of competence:** (a) beer – higher competence of Poland and (b) wine – higher competence of France. Categorization time in milliseconds (ms).

The interaction effects (IAT × product category) was also significant *F*(1,28) = 4.3; *p* = 0.05; η^2^ = 0.14. In case of the beer category the RT was shorter when Polish brands were categorized with positive words and French brands with negative words (*M* = 633; *SD* = 49). When Polish brands were categorized with negative words and French brands with positive words (reversed task) time of categorization was longer *M* = 672; *SD* = 61), *t*(14) = 2.33; *p* < 0.05. When IAT was performed for wine category there was no significant difference whether French or Polish brands were paired with positive or negative words (*M* = 671; *SD* = 73 for [-]FR/PL[+] and *M* = 728; *SD* = 98 for ([-]PL/FR[+]), *t*(14) = 1.69; *p* > 0.1 (see **Figure 4**).

### Discussion

Experiment 3 confirmed once again the existence of ICE – an automatic favoritism of local brands over foreign. Respondents categorized Polish brands faster than French when they were paired at the same side of the computer screen with positive words rather than with negative. This time, when the IAT was conducted on existing brands (and not faked as in Experiment 2) the ICE effect was observed. This result confirmed that the ICE effect is not only caused by the familiarity effect.

Moreover, the results of Experiment 3 showed that automatic and unconscious ICE can be reduced by the country-of-origin effect. In the case of beer brands (where Poland is perceived as more competent) a strong IAT effect in favor of Polish brands was obtained. However, in the case of the wine category the ICE effect was not observed – there was not a significant difference in RT between the two tasks. This effect of minimizing the ICE when the out-group is perceived as a competent producer was obtained only in Experiment 3, where existing brands were used, but not in Experiment 2 – with faked brands.

The results observed in Experiment 3 could have also an alternative explanation. The RTs in the beer IAT were in general shorter than in the wine IAT (this was independent of their origin as either Polish or French). It could be a consequence of the difference in length of the names of beers and the names of wines – in general names of beer brands were shorter. Another explanation for the shorter RT in the case of the beer task could be that, in general, beer brands are much better known than wine brands. The Polish beer brands used in the research were very well-known. The majority of respondents were also familiar with French beer brands. However, the wine brands, both Polish and French, were probably not known. First of all, Poland does not have a wine production tradition and therefore the brands used in the research, even though they were authentic, are very niche products. In the case of French brands of wine, we can suspect that they are also not well-known, because French wines are more recognized by regions (e.g., Bordeaux) or blends (e.g., Merlot) than by brands.

## Implicit Consumer Ethnocentrism (ICE) vs. Competence of Country of Origin (Existing Brands) and the Relationship with Explicit Ethnocentrism – Cetscale (Experiment 4)

The goal of Experiment 4 was to replicate the results obtained in Experiment 3, showing that the competence of the foreign country (out-group) as a producer in some product categories can reduce the automatic ICE effect. However this time we wanted to check the effect for product categories different from those used in Experiments 2 and 3. The categories chosen for Experiment 4 were also beverages, but this time we wanted to choose non-alcoholic drinks and product categories where brands having high awareness. For this purpose we choose juices and sodas.

An additional goal of this study was to investigate the relationship between ICE and explicit measures of consumer ethnocentrism. In Experiment 1 explicit consumer ethnocentrism was measured based on our own method, BPM, but this time we used the classic measure of consumer ethnocentrism – CETSCALE. We wanted to observe the relation between ICE and a popular consumer ethnocentrism measure (CETSCALE).

### Pilot Studies – Choice of Product Categories (Depending on Competence Level), Country of Origin and Names of Existing Brands

The goal of the first pilot study was to check perceived competence of the out-group country (USA) compared to the competence of Poland in two product categories: juices (assumed higher competence of Poland) and sodas (assumed higher competence of USA). Participants (*N* = 30 students) evaluated the competence of Poland and USA in two product categories (juices and sodas) on a seven-point scale. Poland was judged as a more competent producer of juices (*M* = 5.47; *SD* = 1.04) than USA [*M* = 4.87; *SD* = 1.28; *t*(29) = 2.13; *p* < 0.05], USA was perceived as a more competent soda producer (*M* = 5.90; *SD* = 0.88) than Poland [*M* = 4.23; *SD* = 1.41; *t*(29) = 5.34; *p* < 0.001].

The second pilot study was aimed at choosing names of Polish and American brands in two product categories (juices and sodas). Participants (50 students) had to answer two questions referring to each of 24 tested brands: (a) do they know the brand?; (b) do they think the brand is Polish or American? Based on *t*-test analysis for independent samples, brands were chosen which didn’t differ in the level of awareness, and which were clearly recognized as Polish or American. Finally eight brands of juices and eight brands of sodas were chosen (four Polish and four American for each IAT task). The length of the brand names was also controlled. Juices brands chosen for the study were: (a) Polish: Hortex, Smakuś, Sekpol, Siódme niebo; (b) American: Cappy, Clippo, Granini, Donald Duck. Brands chosen for the sodas category were: (a) Polish: Hoop, Zbyszko, Hellena, Polo Cola; (b) American: Schweppes, 7up, Mirinda, Sprite.

### Method

#### Participants

Ninety Polish university students participated in the study, 45 women and 45 men, age 19–26 (*M* = 21; *SD* = 2.4).

#### Research Methods and Materials

Implicit Consumer Ethnocentrism was measured using the IAT method adapted to measure brand preference (as described in previous experiments). The target stimuli used in the study were the names of the Polish or USA brands of juices and sodas chosen in the pilot study. In the study were used two versions of the IAT task, designed to measure ICE in the case of juices and sodas as target categories.

#### Procedure

Each respondent filled in an ICE IAT and consumer ethnocentrism scale (CETSCALE), with half of the respondents completing the IAT first and the other half completing the CETSCALE first. Respondents were also randomly assigned to groups with either juices or sodas as the target category, i.e., half of them accomplished the IAT version with juices as a product category and the other half with sodas.

### Results

To verify the predictions the analysis of variance (mixed mode with repeated measures) was performed: 2 (IAT task [RT]: [-]PL/USA[+] vs. [-]USA/PL[+]) × 2 (product categories: juices vs. sodas). The analysis showed a significant difference in RT between the two IAT tasks (IAT main effect): RT when Polish brands were categorized at the same side of the computer screen with negative words and American with positive ([-]PL/USA[+]) was significantly longer (*M* = 842; *SD* = 130) compared to when the opposite task ([-]USA/PL[+]) was performed [*M* = 766; *SD* = 111; *F*(1,88) = 28.75; *p* < 0.001; η^2^ = 0.25]. This result shows that respondents expressed ICE favoring Polish brands. The effect of product category (juices, *M* = 807, *SD* = 103 and sodas *M* = 800; *SD* = 101) was not significant: *F*(1,89) = 0.43; *p* = 0.5; η^2^ = 0.02.

**FIGURE 5:**
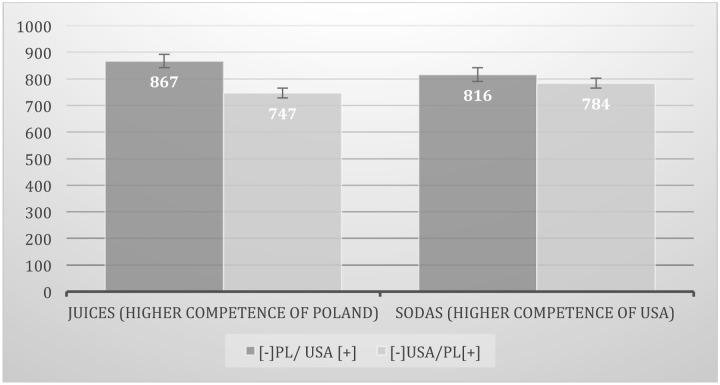
**Comparison of ICE toward Polish (local) vs. American (foreign) existed brands (IAT effect) in two product categories of different level of competence:** (a) juices – higher competence of Poland and (b) sodas – higher competence of USA. Categorization time in milliseconds (ms).

The same analysis of variance showed, however, the significant interaction effect, *F*(1,88) = 9.81; *p* < 0.01; η^2^ = 0.10 (see **Figure 5**). ICE was reduced by competence of country-of-origin. When brands of Polish juices were categorized with positive words and American with negative ([-]USA/PL[+]); *M* = 747; *SD* = 108) RTs were significantly shorter than when Polish juices brands were categorized with negative words and American with positive ([-]PL/USA[+]; *M* = 867; *SD* = 148; *t*(44) = 4.21; *p* < 0.001). In the category when Poland was a competent producer (juices) participants expressed ICE. However, in the category where the out-group was a competent producer and Poland was inferior (sodas) there were not significant differences between IAT tasks (ICE was not observed – [-]USA/PL[+]; *M* = 784; *SD* = 111; [-]PL/USA[+]; *M* = 816; *SD* = 104; *t* < 1, *p* > 0.1). Both results confirmed predictions.

Explicit consumer ethnocentrism measured with CETSCALE did not show a significant correlation with ICE as measured by IAT (*r* = 0.1; *p* > 0.1).

### Discussion

Experiment 4 replicated the evidence for the existence of ICE, this time in the context of a new product category: juices and sodas, and in the context of a new country-of-origin (out-group) – USA. The results of this study confirmed also that ICE could be reduced when foreign products are associated with higher competence than local products (as in case of sodas). However, ICE could be weakened or reduced only in the case of existing and well-known brands. It suggests that implicit in-group favoritism (the mechanism underlying the ICE effect) can be modified only by the creation of other strong automatic associations (as observed in the case of well-known brands).

The analysis didn’t show a significant correlation between two measures of consumer ethnocentrism: CESCALE and IAT. One of the explanations could be the difference in what each method is measuring, and the different origins of both constructs. ICE could be formed (as suggested by data from the experiments conducted) based on automatic preference for objects associated with the in-group – in this case locally manufactured products. In turn, explicit ethnocentrism (measured by CETSCALE) could be formed based on more rational prerequisites and moral beliefs that local industry should be supported. This is consistent with Shimp and Sharma’s (1987) assumption that (explicit) consumer ethnocentrism has a moral and ideological background.

## General Discussion

### Consumer Ethnocentrism and Implicit Consumer Ethnocentrism (ICE)

Consumer ethnocentrism, defined as a conscious preference for products from one’s own country as opposed to foreign products, in our study was measured using: (a) the classic consumer ethnocentrism scale CETSCALE and (b) BPM (Brand Preference Measure) – a method based on forced choice of one used or preferred brand from a pair of brands belonging to the same category. The results from both explicit measures demonstrated limited consumer ethnocentrism among Poles. This was particularly evident when the BPM (Experiment 1) had been used, where half the respondents picked only foreign brands as the brands they used or preferred in all the categories. This once again confirms that outside the US, particularly in less developed countries (now or in the recent past), consumers are significantly less ethnocentric.

The results of the four conducted studies provided insight into a new aspect of consumer ethnocentrism, that it might also have an automatic character. ICE was observed for existing brands (study 1); for non-existent brands (study 2); for different product categories: wines and beers (studies 2, 3), juices and sodas (study 4); and mixed categories (study 1). To measure ICE an adaptation of the IAT method was used. However, because of the results obtained some questions have arisen. The first is to do with whether the IAT measurements presented in the studies demonstrate a product-related attitude (ICE) or whether they result from a simple categorization into “domestic” vs. “foreign” (with products not being considered), which in fact is manifested in words related to Poland (or Polish-sounding words) being more easily categorized with positive words rather than with words not related to Poland. If this were to be solely a reflection of a “domestic” vs. “foreign” categorization, the presented findings would not be the result of consumer attitudes and would only stem from a purely psychological mechanism of categorization. However, if the IAT measurement were to only reflect a purely psychological categorization mechanism (bypassing product-related attitudes), then it would have to be identical regardless of whether Polish products were perceived as superior or inferior in quality. This was not the case. The findings of studies 3 and 4 demonstrate that if a given country (out-group) is seen to be a competent producer, then this implicit preference for the in-group is reduced. Thus the hypothesis that argued that the ICE’s effect is only linguistic in nature has been refuted. The studies also ruled out the existence of another, alternative explanation of the ICE effect: that it had been obtained based only on the familiarity effect (Polish brand names being more familiar than out-group brand names). The ICE effect has also been observed when awareness of domestic and foreign brands was controlled (in Experiment 4). With the awareness of juice and soda brands by Polish and American manufacturers being identical, the IAT effect was significant only for those Polish brands, which were part of the highest competence category (juices). The findings of these studies serve not only as a source of data on how to measure consumer ethnocentrism, but also facilitate the understanding of this phenomenon. It is not one-dimensional and includes both automatic and reflective components. The consumer ethnocentrism phenomenon is not only, as Shimp and Sharma (1987) suggested, an ideology-based conscious mechanism, but is also based on automatic mechanisms.

Implicit Consumer Ethnocentrism can be explained by a psychological mechanism of in-group favoritism. This mechanism implies that a product manufactured in one’s own country immediately gets “credit points” for its origin and is automatically perceived in a more favorable way by the consumer. However, a given individual’s attitude doesn’t always have to be consistent across all the measurements and an individual doesn’t always demonstrate just one attitude toward one object. Automatic and unconscious beliefs about a product might be different from the conscious, more reflective ones: while on an implicit level, a given individual may prefer “domestic” products and his/her attitude might be clearly ethnocentric, on a more reflective level it might be more internationally oriented, based on a conviction that local manufacturers are less technologically savvy than multinational corporations.

### Implicit Consumer Ethnocentrism Can be Reduced by Country of Origin Effect

The second goal of the project was to investigate whether ICE – a strong, automatic mechanism – can be limited by a competitive effect (competence of country of origin). Traditionally it had been assumed that the country-of-origin effect may arise from rational, affective or normative premises (Obermiller and Spangenberg, 1989; Verlegh and Steenkamp, 1999). As already mentioned, information about a product’s country of origin affects information processing (Hong and Wyer, 1989; Maheswaran, 1994). Whether or not directly disclosed, information about the country of origin that’s desirable in a given product category instantly gives us more confidence in a brand. The mechanism behind how the country-of-origin information works is similar to how stereotypes work. Just as stereotypes manifest themselves automatically (Greenwald and Banaji, 1995), so can the country-of-origin effect. Gürhan-Canli and Maheswaran (2000) have found that individuals with greater motivation to provide a correct response formulate their opinions about a product based on the country-of-origin information to a lesser extent and tend to pay more attention to other sources of information.

Two interrelated automatic behaviors, such as implicit ethnocentrism and the country-of-origin effect, can drive the choice of a product. Because of the basic nature of the preference for “domestic” products, it seems that consumer ethnocentrism is a strong mechanism. Meanwhile, the country-of-origin effect may limit its impact on product preferences, depending on how strong and powerful the newly created automatism related to the country-of-origin is. Those assumptions were proved in this research project. The results of study 4 showed that the country-of-origin can reduce automatic in-group favoritism. This effect was observed for well-known brands that people were experienced with (study 4). But it was not observed in study 2 for unknown and faked brands. Also in study 3, where lesser known wine brands were used, the impact of the country of origin was insignificant. These findings suggest that information about the country of origin only influences ICE when it is based on strong, automatic associations.

### Limitations of the Study

The findings obtained in the studies presented contribute to a broader discussion about changing implicit attitudes. For a long time researchers expressed skepticism as to whether implicit attitudes may in fact be subject to change. Their skepticism was rooted in the belief, that implicit attitudes are in fact traces of past experiences which don’t change easily. Nevertheless, numerous experiments have been conducted in recent years which proved that such a change is possible (e.g., Wittenbrink et al., 1997; Blair et al., 2001; Dasgupta and Greenwald, 2001; Karpinski and Hilton, 2001; Rudman et al., 2001; Dijksterhuis, 2004; Gawronski and Bodenhausen, 2006). Gawronski and Bodenhausen (2006) assume that a change in an implicit attitude, and more precisely a modification of that attitude, may be an expression of a *temporary increase in the availability of certain association patterns* (e.g., African Americans = family people). Hence, we are not dealing with one attitude but rather with different association patterns connected to a given object. The study of Wittenbrink et al. (1997) showed that implicit attitudes toward African Americans can be modified depending on whether the group is shown in a positive (family) or negative (violence) context. After being exposed to African Americans in a positive light, subjects’ implicit attitudes toward African Americans were less negative than when they were shown in a negative light. Even though our study dealt with a completely different subject area, analogous mechanisms have been observed. On an automatic level consumers have a preference for domestic products. Nevertheless, such a preference may be modified when faced with a product, which is connected with a different association, e.g., foreign as better/superior. This is consistent with the assumption of Bargh (1994) that the only effective way of restricting automatisms is to activate a competitive automatism. However, as the findings of Experiment 4 have demonstrated, this must be a strong automatic association.

Two of the conducted studies explored the relation between ICE and explicit measures (CETSCALE and BPM). Especially, observing the discrepancy between implicit and explicit measures of ethnocentrism an interesting question for further studies might be the relation of explicit and ICE with real product choice. It is a question about predictive value of the ICE comparing to explicit measures. However, it strongly depends on the type of explicit measure used, weather it is more ideology or cognition based or more affect based. Probably explicit measures, which rely more on affect than cognition will be more related to an implicit measure of consumer ethnocentrism.

Another question is connected to the purchase situation: how big a role does consumer ethnocentrism play in the buying decision, compared to other factors as product features, it’s objective quality, price, availability and brand awareness or brand image? Unfortunately, already conducted studies cannot answer this question, because they were concerned with the exploration of only one factor – consumer ethnocentrism and the mechanisms underlying it. Nevertheless the role of consumer ethnocentrism in the whole buying decision process is worth exploring in future studies.

### Application

Results from current studies indicate implicit ethnocentrism and explicit ethnocentrism are two different phenomena. Dissociation of explicit and implicit ethnocentrism is particularly likely in less developed countries, where foreign brands are preferred and bought more often than their domestic alternatives and a discrepancy between implicit and explicit attitudes may occur. It is also likely to appear in former Eastern Block countries, where independently of current economic development, past experience taught people that local products have inferior quality. In such circumstances a person may experience an internal clash between an implicit attitude based on an automatic, emotional preference (domestic seen as better than foreign) and an explicit, rational judgment based on observation and experience (foreign is better). Consumers with a high level of consumer ethnocentrism have a dilemma because they’re faced with a choice of whether to buy a local or “domestic” product which they view as inferior, or a foreign product which they view as superior. They have a dilemma as to whether to buy “ours but worse” or “theirs but better.”

The confrontation of two other phenomena: *consumer ethnocentrism* and *the-country-of-origin effect* has important application-related implications since this is what marketing departments deal with on a daily basis. When launching a new foreign brand to the market, brand managers must decide whether to highlight in communication the fact of it’s origin. There is no clear-cut solution to this problem, because as research discussed here has shown, it will depend on many factors, such as the product category, the country of origin, and the public perception of that country’s competence in manufacturing products from that category. It may be assumed that if these are low-engagement, impulse-purchased products producers may claim their ingredients are sourced from the domestic market (e.g., milk in yogurt) and thereby create jobs for the citizens of the country the consumer lives in. It’s a good idea to communicate this local character. As a result, the producer is creating locally integrated local brands (Kipnis et al., 2012). Alternatively, when a producer is seen to possess a high level of competence in a given product category, he may accentuate the product’s foreign origin, thus boosting the likelihood that it will achieve success in the new local market because the local population perceives that foreign products (from this particular category) are better.

## Author Contributions

Conceived and designed the experiments: NM, DM. Performed the experiments: NM, DM. Analyzed the data: NM, DM. Wrote the paper: DM, NM.

## Conflict of Interest Statement

The authors declare that the research was conducted in the absence of any commercial or financial relationships that could be construed as a potential conflict of interest.
